# Implications of the introduction of laboratory demand management at primary care clinics in South Africa on laboratory expenditure

**DOI:** 10.4102/ajlm.v5i1.339

**Published:** 2016-03-18

**Authors:** Ozayr H. Mahomed, Ruth Lekalakala, Shaidah Asmall, Naseem Cassim

**Affiliations:** 1 Discipline of Public Health Medicine, University of KwaZulu-Natal, Durban, South Africa; 2 Clinical Microbiologist, University of Limpopo, Polokwane, South Africa; 3 Senior Technical Advisor, National Department of Health, Pretoria, Gauteng, South Africa; 4 Data and Logistics Manager, National Health Laboratory Service, Johannesburg, Gauteng, South Africa

## Abstract

**Background:**

Diagnostic health laboratory services are regarded as an integral part of the national health infrastructure across all countries. Clinical laboratory tests contribute substantially to health system goals of increasing quality of care and improving patient outcomes.

**Objectives:**

This study aimed to analyse current laboratory expenditures at the primary healthcare (PHC) level in South Africa as processed by the National Health Laboratory Service and to determine the potential cost savings of introducing laboratory demand management.

**Methods:**

A retrospective cross-sectional analysis of laboratory expenditures for the 2013/2014 financial year across 11 pilot National Health Insurance health districts was conducted. Laboratory expenditure tariff codes were cross-tabulated to the PHC essential laboratory tests list (ELL) to determine inappropriate testing. Data were analysed using a Microsoft Access database and Excel software.

**Results:**

Approximately R35 million South African Rand (10%) of the estimated R339 million in expenditures was for tests that were not listed within the ELL. Approximately 47% of expenditure was for laboratory tests that were indicated in the algorithmic management of patients on antiretroviral treatment. The other main cost drivers for non-ELL testing included full blood count and urea, as well as electrolyte profiles usually requested to support management of patients on antiretroviral treatment.

**Conclusions:**

Considerable annual savings of up to 10% in laboratory expenditure are possible at the PHC level by implementing laboratory demand management. In addition, to achieve these savings, a standardised PHC laboratory request form and some form of electronic gatekeeping system that must be supported by an educational component should be implemented.

## Introduction

Diagnostic health laboratory services are regarded as an integral part of the national health system across all countries and have an important role in the continuum of care. Laboratory testing provides access to screening of asymptomatic individuals at risk for developing disease, early detection of diseases and diagnostic confirmation; provides information on patients’ prognosis; assists with planning appropriate disease management strategies and monitoring patients’ response to treatment; and plays a pivotal role in ensuring patient safety by identifying hospital-acquired infections and other potential health related adverse events.^1^

The National Health Laboratory Service (NHLS) provides diagnostic laboratory services for the South African public health sector through over 300 laboratories across the nine provinces, thereby achieving 80% population coverage.^2^ The NHLS is reimbursed by the Provincial Departments of Health on a fee-for-service billing arrangement.^2^ Through this payment mechanism, laboratory tests are itemised as tariff codes on an invoice, for example, tariff code 2210 denotes the haemoglobin test. These funds are obtained from the provincial equitable share portion of the national health budget.

Expenditures for laboratory services have increased by 45% from R3.1 billion South African Rand in financial year (FY) 2010/2011 (01 April 2010 to 31 March 2011) to R4.5 billion by FY 2013/2014 (01 April 2013 to 31 March 2014).^3^ This was because of increases in laboratory test volumes from 80.2 million tests in FY 2011/2012 to 81.1 million in FY 2012/2013^4^ and approximately 86 million tests in FY 2013/2014.^2^ The sharp increases in expenditures and test volumes in FY 2013/2014 can be attributed to growth in priority test volume. Priority tests for patients living with HIV, tuberculosis and cervical cancer accounted for 16% of total test volume^2^ in FY 2013/2014. There was a 144% increase in the volume of GeneXpert MTB/RIF assays, as well as a 27% increase in HIV viral load testing.^3^ The GeneXpert MTB/RIF assay replaced the auramine smear for diagnosis of tuberculosis during this time period. There was a large difference in the price per test; auramine smears cost R24.34, whereas the GeneXpert MTB/RIF assay cost R172.85 as of December 2013.

The volume of laboratory testing is anticipated to increase with the introduction of general practitioners as care providers for patients at the primary healthcare (PHC) level, because general practitioners are expected to order additional laboratory tests that were not previously requested under nurse-based PHC services. Furthermore, a renewed focus has been placed on integrated clinical services at the PHC level^5^ and the introduction of algorithms to appropriately manage patient conditions.^6^ This is likely to further increase the volume of tests requested, thereby increasing the total expenditures of the public health system.

Demand management aims to improve the requesting of appropriate laboratory tests and results in reductions in public health expenditures without affecting clinical outcomes.^7^ The first step to implementing demand management involves defining what constitutes an ‘inappropriate’ request, based on some form of agreed-upon guidance.^7^ For example, this may involve standardising the repertoire of tests that may be requested by level of care, namely, PHC and hospital services.^7^ Similarly, evidence-based laboratory medicine involves eliminating laboratory tests with no clinical value and introducing new laboratory tests where evidence proves their efficacy and effectiveness.^8^ The implementation of this approach requires a pathologist-driven laboratory service that utilises context-appropriate evidence to guide testing and reduce public health expenditures.

The South African National Department of Health has proposed a demand-management system in the form of an essential laboratory tests list (ELL) to promote appropriate and cost effective usage of laboratory services at PHC facilities without having a negative effect on patient outcomes. The ELL includes the minimum set of tests that should be performed to offer comprehensive services at the PHC level.^9^ In determining the ELL for South Africa, the World Health Organization criteria for the usefulness and clinical relevance of tests that influence diagnosis and patient management were considered.^9^ Additionally, the ELL requires that a single test be used, rather than multiple tests, if the single test provides adequate diagnostic information.^9^ For example, on the ELL, the alanine transaminase test is substituted for the liver function panel test, as the alanine transaminase test provides the same diagnostic value without affecting patient outcomes. Furthermore, the laboratory tests included on the ELL were aligned to the national South African standard treatment guidelines,^10^ including the PHC clinical algorithms that were introduced as a clinical supportive management component of the integrated chronic disease management mode.^5^

The aims of this study were to analyse current expenditures and the profile of laboratory tests currently requested at the PHC level. In addition, we sought to determine the potential cost savings that could be achieved by the public health sector through the introduction of the demand-management-based ELL.

## Research method and design

This was a retrospective cross-sectional analysis of PHC laboratory expenditure for the FY 2013/2014 period across 11 National Health Insurance (NHI) pilot districts. Data on district and facility level expenditures for the Amajuba, City of Tshwane Metro, Dr Kenneth Kaunda, Eden, Gert Sibande, OR Tambo, Pixley ka Seme, Thabo Mofutsanyana, Umgungundlovu, Umzinyathi and Vhembe districts were extracted from the NHLS Corporate Data Warehouse. The extracted data fields included customer account information, laboratory information, system location codes used to identify health facilities, tariff codes used for billing purposes (to identify the investigation(s) performed), annual test volumes and expenditures. All expenditure data were reported in South African Rand.

The ELL defines the laboratory tests that could be requested at PHC facilities by nursing staff or general practitioners. For example, nurses can request thyroid stimulating hormone tests; however, general practitioners can also request a free thyroxine 4 test. Amongst other tests, the ELL included haemoglobin, HIV viral load, GeneXpert for tuberculosis, cluster definition 4 (CD4) count, HIV DNA PCR for infants, sputum and urine microscopy, smear, culture and sensitivity, total cholesterol and total triglycerides, prostatic specific antigens, cervical smears and glycated haemoglobin (HbA1c). Tests not on the ELL for primary healthcare included full blood count, urea and electrolytes, HIV serology, liver function tests, drug levels for carbamezapine and tegretol, endocrine tests such as thyroid profiles and follicule stimulating hormones and luteinising hormone levels, arthritis screening, anti-nuclear factors and other immunological tests.

Each ELL test was mapped to one or more NHLS tariff code(s) from the expenditure data. A one-to-one or a one-to-many relationship exists between an ELL test and the tariff code(s) used by the NHLS. For example, the CD4 test represents a one-to-one relationship. However, the C-reactive protein test has a one-to-many relationship, as different tariff codes are used based on the laboratory methodology, for example, qualitative versus quantitative test. Furthermore, some ELL tests follow a diagnostic cascade, whereby based on an initial result, a subsequent investigation is performed, such as microscopy, culture and sensitivity. Additionally, ELL tests were grouped into logical test baskets, for example, lipogram for cholesterol and triglycerides.

For the expenditure analysis, a Microsoft Access 2010 database (Microsoft Corporation, Redmond, Washington, United States) was used and the expenditures and ELL mapping list were imported as tables. The expenditure tariff codes were reported with the ELL test by creating a relationship between the two tables. Queries were developed to report on tariff code expenditures based on the ELL test.

## Results

### Test volumes and expenditures by health district

Approximately 4.5 million tests accounted for approximately R339 million in laboratory expenditures for diagnostic laboratory tests at the PHC facilities in the 11 districts during the FY 2013/2014 study period (Table 1). PHC facilities within the City of Tshwane (Gauteng) accounted for the highest proportion of test volume and laboratory expenditures, followed by clinics within the OR Tambo district (Eastern Cape) and Umgungundlovu district (KwaZulu-Natal).

**TABLE 1:** Test volumes and laboratory expenditures for primary healthcare facilities within 11 National Health Insurance pilot districts, South Africa, 2013–2014.†

National Health Insurance pilot district	Number of tests	Percentage of total number of tests (%)	Expenditure (ZAR)	Percentage of total expenditure (%)
Amajuba	204 014	5	R16 957 104	5
City of Tshwane Metro	960 437	22	R70 207 965	21
Dr Kenneth Kaunda	385 380	9	R30 002 610	9
Eden	286 446	6	R18 725 978	6
Gert Sibande	481 014	11	R34 079 371	10
OR Tambo	544 269	12	R42 377 609	12
Pixley Ka Seme	161 371	4	R11 449 909	3
Thabo Mofutsanyana	337 386	8	R31 073 208	9
Umgungundlovu	484 247	11	R42 361 325	12
Umzinyathi	158 848	4	R12 123 605	4
Vhembe	443 529	10	R29 958 630	9
**Total**	**4446 941**	**100**	**R339317 313**	**100**

ZAR, South African Rand.

† Analysis included data for financial year 2013/2014, which began 01 April 2013 and ended 31 March 2014.

### Total expenditures on ELL and non-ELL laboratory tests

Of all laboratory expenditures for FY 2013/2014, 21 tests were responsible for ~92% (R310 million) of the total (Table 2). Laboratory tests for patients living with HIV were responsible for ~47% of all expenditures, of which HIV viral load accounted for ~32% of expenditures, CD4 for ~10% and HIV DNA PCR for infants, ~6%. Laboratory tests for tuberculosis diagnosis accounted for ~21% of the expenditure, including tuberculosis microscopy (~2%), GeneXpert (~18%) and tuberculosis culture (~1%). Non-ELL tests such as the full blood count and the urea and electrolyte tests were responsible for 5% of expenditure (R16 million).

**TABLE 2:** Laboratory tests with the highest expenditures across the 11 National Health Insurance pilot districts, South Africa, 2013–2014.†

Test description	Expenditure (ZAR)	Percentage of total expenditure‡ (%)	Cumulative percentage§ (%)
HIV viral load	107 094 208	31.6	31.6
GeneXpert for MTB/RIF	61 937 837	18.3	49.9
CD4	33 685 531	9.9	59.8
HIV DNA PCR	19 646 244	5.8	65.6
Creatinine	15 596 873	4.6	70.2
Full blood count¶	9 807 196	2.9	73.1
Alanine transaminase	9 136 749	2.7	75.8
Urea and electrolytes¶	6 989 505	2.1	77.9
Pap smear	6 668 283	2.0	79.9
Tuberculosis direct (auramine)	5 939 449	1.8	81.7
Hepatitis A IgG	5 027 926	1.5	83.2
Rapid plasmin reagin	4 888 999	1.4	84.6
Tuberculosis culture	4 242 050	1.3	85.9
Rhesus factor¶	3 629 905	1.1	87.0
Cholesterol	3 191 403	0.9	87.9
Haemaglobin	2 935 956	0.9	88.8
Thyroid stimulating hormone	2 597 554	0.8	89.6
Glycated haemoglobin	2 110 422	0.6	90.2
Tryglyceride	1 967 680	0.6	90.8
Aspartate transaminase¶	1 805 915	0.5	91.3
Prostatic specific antigen	1 764 142	0.5	91.8
**Total**	**310 663 827**	**91.8**	**91.8**

MTB/RIF, *Mycobacterium tuberculosis*/Rifampicin; IgG, Immunoglobulin G; ELL, essential laboratory list; PHC, primary healthcare; ZAR, South African Rand.

† Analysis included data for all laboratory tests (both tests on the ELL and tests not on the ELL) billed in financial year 2013/2014, which began 01 April 2013 and ended 31 March 2014;

‡ Percentage of total laboratory expenditure (R339 317 313);

§ Cumulative percentage of total expenditure;

¶ These tests were not listed in the ELL for PHC services and were thus considered ‘inappropriate’.

### Expenditure on non-ELL tests by district

Across the 11 NHI pilot districts, 10% (R35 million) of laboratory expenditures at PHC facilities were for tests that were not included on the ELL (Table 3). In five districts, including Gert Sibande, Pixley Ka Seme, Vhembe, City of Tshwane and Dr Kenneth Kaunda, the proportion of tests not included on the ELL exceeded 10% of the PHC’s laboratory expenditure.

**TABLE 3:** Laboratory expenditures for essential laboratory list (ELL) tests and non-ELL tests within the 11 National Health Insurance pilot districts, South Africa, 2013–2014.†

NHI pilot district	ELL test expenditure (ZAR)	ELL percentage of district total expenditure (%)	Non-ELL test expenditure (ZAR) (%)	Non-ELL percentage of district total expenditure (%)	Total district expenditure (ZAR)
Amajuba	15 703 295	93	1 253 809	7	16 957 104
City of Tshwane Metro	61 977 231	88	8 230 733	12	70 207 965
Dr Kenneth Kaunda	26 806 032	89	3 196 577	11	30 002 610
Eden	16 934 366	90	1 791 612	10	18 725 978
Gert Sibande	29 460 294	86	4 619 077	14	34 079 371
OR Tambo	38 710 524	91	3 667 085	9	42 377 609
Pixley Ka Seme	9 944 644	87	1 505 265	13	11 449 909
Thabo Mofutsanyana	29 494 934	95	1 578 274	5	31 073 208
Umgungundlovu	37 962 309	90	4 399 017	10	42 361 325
Umzinyathi	10 976 833	91	1 146 772	9	12 123 605
Vhembe	26 309 844	88	3 648 786	12	29 958 630
Total expenditures	304 280 306	-	35 037 007	-	339 317 314
**Totalpercentage of expenditures (%)**	**90**	**-**	**10**	**-**	**100**

NHI, National Health Insurance; ELL, essential laboratory list; ZAR, South African Rand.

† Analysis included data for all laboratory tests (both tests on the ELL and tests not on the ELL) billed in financial year 2013/2014, which began 01 April 2013 and ended 31 March 2014.

### Profile of the non-ELL tests processed

**FIGURE 1:**
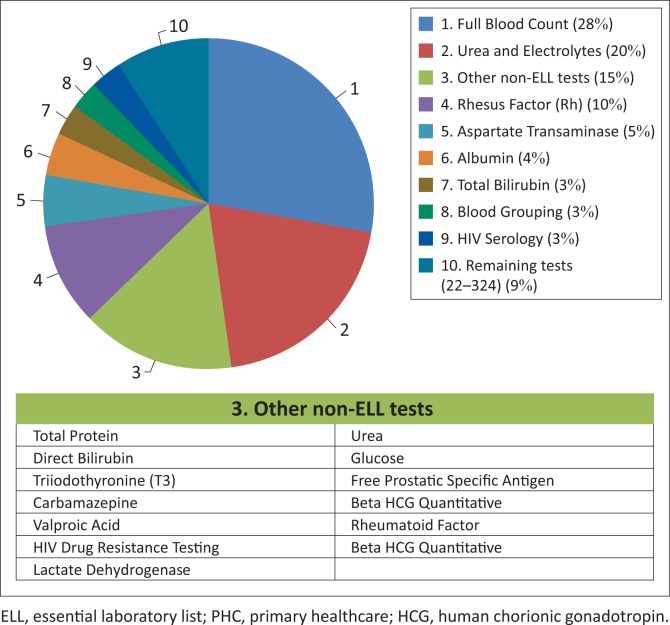
Non-ELL expenditures within the 11 National Health Insurance pilot districts, South Africa, 2013−2014. Analysis included data for the 21 most common tests not on the ELL that were billed in financial year 2013/2014, which began on 01 April 2013 and ended 31 March 2014. These 21 tests represented 91% of ‘inappropriate’ (non-ELL) laboratory expenditures for the PHC facilities within the 11 NHI pilot districts.

Of all the laboratory tests not included on the ELL, 21 tests accounted for 91% (R31 million) of the total non-ELL laboratory expenditures (R35 million) (Figure 1). Of these tests, the full blood count (28%) and urea and electrolyte (20%) tests were the main cost drivers for non-ELL tests. The third main contributor was the different components of the liver function test, which together accounted for 17% of expenditures for non-ELL tests (aspartate transaminase, 5%; total protein, 2%; albumin, 4%; total bilirubin, 3%; direct bilirubin, 2%; and lactate dehydrogenase, 1%). Rhesus factor laboratory tests accounted for 10% of the non-ELL laboratory expenditures. The remaining non-ELL tests accounted for 9% of non-ELL expenditure.

## Discussion

This retrospective analysis of laboratory expenditure data for FY 2013/2014 indicated that facilities within the 11 NHI pilot districts accounted for approximately R339 million of all PHC facility expenditures for diagnostic laboratory tests. The City of Tshwane had the highest proportion for test volume and laboratory expenditure, followed by facilities within the OR Tambo and Umgungundlovu districts. Diagnostic tests for HIV and tuberculosis were the main cost drivers for laboratory expenditure. Of the estimated R339 million total, approximately R35 million (10%) were for non-ELL tests. Full blood count, urea and electrolyte profiles, as well as liver function tests usually done to support the holistic management of patients on ART, were the main cost drivers for non-ELL tests.

Public health expenditures overall are expected to increase by an average of 7.9% between FY 2014/2015 and FY 2016/2017.^6^ Laboratory expenditures are expected to increase by an average of 17.7% during the same time period. This is likely to increase budgetary pressures on an already cash-strapped public health sector and, in particular, on the NHLS. However, despite these financial constraints, the NHLS is expected to provide or maintain the same standard of service.

Laboratory, patient, healthcare provider and systemic factors are often cited as potential reasons for ‘inappropriate laboratory tests requests’.^7^ Laboratory factors include prolonged turn-around-times, inability to access results due to the lack of information systems, laboratory request forms that enable the request of a panel test rather than an individual test and the availability of an open-ended, ‘other tests’ box.^7^ Healthcare providers may inappropriately request tests because of inexperience, inadequate understanding or lack of awareness regarding the guidelines or protocols of management, a lack of information about the unit cost of each test, or routine practice.^11^ Additionally, the poor filing systems within health facilities often result in duplicate test requests.^7^

In 2008, the Carter Review noted that 25% of pathology tests conducted in the United Kingdom National Health Service were unnecessary^7^ and the implementation of demand management would conservatively result in a 20% savings in laboratory expenditures.^12^ Our study found that an average of 10% of the laboratory test expenditures at the PHC level were for non-ELL tests. If use of the demand-management-based ELL were extended to district, regional and tertiary hospitals, the savings would be in line with the Carter estimate. A conservative estimate based on the 10% savings applied to all 3400 PHC facilities across 52 NHLS districts would result in an annual savings of R400 million to the health sector.

## Recommendations

Currently, laboratories are required to perform all tests requested by the clinician and/or nursing staff, resulting in over-utilisation of services. To address inappropriate use of laboratory tests (i.e., non-ELL tests) across South Africa by PHC facilities, three key initiatives are proposed.

The first initiative is the development of a national ELL. This would require the standardisation of typical clinical laboratory tests per level of healthcare, whilst taking into consideration local demographic and epidemiological factors.^9^ The second initiative would be to support the ELL by developing a dedicated PHC laboratory request form that lists only tests appropriate for the PHC level. Clinicians and nurses would thus be able to select only from amongst tests on the ELL. An important aspect of the PHC request form design would be to remove the ‘other tests’ box, which enables clinicians to request any investigation. The first two initiatives should, in turn, be supported by the third: electronic gatekeeping to reject tests that are inappropriately requested (not on the ELL). However, in order to achieve this initiative, all PHC health facilities would be required to use a health information system, including an order entry module with built-in rules to avoid inappropriate ordering.

Whilst the above measures may help to reduce inappropriate laboratory test requests, appropriate education initiatives directed at health service providers would also be required to support these interventions. These educational sessions should provide guidance on appropriate laboratory testing based on clinical guidelines and evidence-based laboratory medicine recommendations to ensure that specimens are collected in the correct manner.^7^

### Limitations

This study was a retrospective cross-sectional study and used secondary data on expenditures; it was thus dependent on the accuracy of the data entered into the information systems. It was not possible to differentiate whether tests were coded accurately or combined when multiple individual tests were ordered. In addition, the study was limited to NHI health districts and the results may not be representative of other health districts in South Africa. Finally, the study focused on one aspect for potential savings. Additional studies may be required to investigate other aspects of appropriate utilisation of the diagnostic laboratory services.

## Conclusion

This study demonstrated that considerable potential savings of up 10% in laboratory expenditure are possible following the introduction of an ELL at the PHC level, in addition to further laboratory demand management interventions.
